# An Effort to Isolate *Mycobacterium bovis* from Environmental Substrates during Investigations of Bovine Tuberculosis Transmission Sites (Cattle Farms and Wildlife Areas) in Michigan, USA

**DOI:** 10.5402/2011/787181

**Published:** 2011-09-22

**Authors:** Amanda E. Fine, Daniel J. O'Brien, Scott R. Winterstein, John B. Kaneene

**Affiliations:** ^1^Center for Comparative Epidemiology, Department of Large Animal Clinical Sciences, College of Veterinary Medicine, Michigan State University, East Lansing, MI 48824-1314, USA; ^2^Wildlife Conservation Society, Mongolia Country Program, P.O. Box 485, Post Office 38, Ulaanbaatar 211238, Mongolia; ^3^Wildlife Disease Laboratory, Michigan Department of Natural Resources, 4125 Beaumont Road, Lansing, MI 48910-8106, USA; ^4^Department of Fisheries and Wildlife, College of Agriculture and Natural Resources, Michigan State University, 13 Natural Resources, East Lansing, MI 48824, USA

## Abstract

Deer movements on cattle farms, wildlife feeding, and livestock management practices in Michigan are thought to create opportunities for indirect transmission of *Mycobacterium bovis* via environmental substrates. To confirm the presence of viable *M. bovis* in the environment, substrates were collected from 13 farms with culture-confirmed *M. bovis* in cattle and 5 sites with high prevalence of *M. bovis* in free-ranging deer. None of the samples processed for mycobacterial culture were positive for *M. bovis*. Agent, host, and landscape-level factors decrease the probability of detecting *M. bovis* in the environment using conventional mycobacterial culture. Molecular techniques that increase the probability of *M. bovis* detection in environmental substrates should be applied to known sites of *M. bovis* transmission in Michigan. In the interim, epidemiological investigations informed by experimental studies will be most effective in characterizing *M. bovis* persistence in the environment and its role in the indirect interspecies transmission of *M. bovis*.

## 1. Introduction

The State of Michigan lost its United States Department of Agriculture designation as “Free from Tuberculosis (TB)” in 2000. This reversal of the State's TB Free status, originally achieved in 1979, was the result of the detection of bovine TB in Michigan cattle in 1998 and confirmation of the establishment of bovine TB in free-ranging white-tailed deer (*Odocoileus virginianus*) in northeast lower Michigan in 1995 [1]. Fifty farms with *Mycobacterium bovis *infected cattle have been detected in Michigan since intensive surveillance for TB in livestock was reinitiated in 1998 [2].

The occurrence of bovine TB in cattle and white-tailed deer in Michigan has a similar temporal and spatial distribution [3]. In addition, DNA fingerprinting techniques have revealed that cattle and deer are infected with an identical strain of *M. bovis *[4]. Evidence supports interspecies transmission of *M. bovis *from the wildlife reservoir (white-tailed deer) to cattle which is assumed to occur through indirect means via shared feed [5]. White-tailed deer are the presumed source of *M. bovis *infection in cattle in over 50% of the herds identified as TB-positive in Michigan [6]. Disease transmission in these instances is thought to occur in the absence of close contact between cattle and deer, since nose-to-nose contact between the two species is rarely observed [7]. 

Bovine TB in Michigan's white-tailed deer population has been characterized as having an endemic focus within the five-country area of Presque Isle, Montmorency, Alpena, Oscoda, and Alcona counties, where 97% of the TB-positive deer have been found [6, 8, 9]. The detection of bovine TB in Michigan cattle has been concentrated in the same area of Michigan. This region of the State, encompassing the five counties that make up the endemic focus of bovine TB in deer in addition to Antrim, Charlevoix, Cheboygan, Crawford, Emmet, Otsego, and portions of Iosco and Ogemaw counties have been designated as “infected” with bovine tuberculosis and classified as “modified accredited” under the guidelines of the Federal Bovine Tuberculosis Eradication Uniform Methods and Rules [10]. 

The overall goal of this study was to perform targeted sampling of environmental substrates in known bovine TB transmission sites and apply sample processing procedures developed for processing environmental samples for *M. bovis *detection in an effort to characterize the persistence of *M. bovis *in the environment and the role of indirect transmission of *M. bovis *in the epidemiology of bovine TB in Michigan. The study attempted to detect *M. bovis *in the environment and determine whether or not it could survive for sufficient lengths of time to serve as a source of infection for cattle and/or wild deer. The work compliments that published by Witmer et al. [11] which focused on sampling a range of wildlife species found on farms and in wildlife areas in the bovine TB outbreak region of Michigan in an attempt to identify a wildlife reservoir for *M. bovis *other than white-tailed deer. 

## 2. Materials and Methods

### 2.1. Identification of Bovine Tuberculosis Transmission Sites

**Figure 1:**
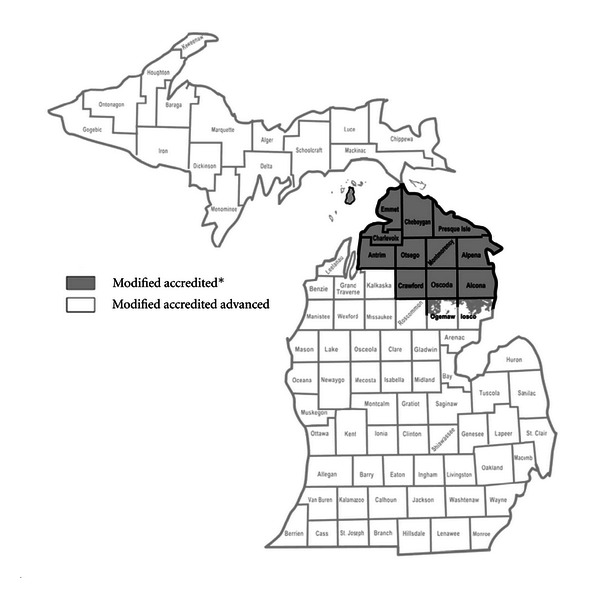
Map of Michigan indicating the Bovine Tuberculosis State Status Designations: (1) modified accredited (infected zone) and (2) modified accredited advanced (disease-free zone). Source: Michigan Department of Agriculture, http://www.michigan.gov/bovinetb.

Potential sites of bovine TB transmission were defined as Michigan cattle farms with confirmed *M. bovis* infection or Michigan townships with the highest recorded apparent prevalence of *M. bovis* in free-ranging white-tailed deer. Bovine TB-positive cattle farms were those farms with a culture-confirmed case of *M. bovis *infection identified through the State and Federal bovine TB surveillance program. Cattle farms identified as bovine TB-positive were presumed to be sites of bovine TB transmission. All of the farms investigated were located in the region of the State of Michigan designated as “infected” with bovine tuberculosis and classified as “modified accredited” under the guidelines of the Federal Bovine Tuberculosis Eradication Uniform Methods and Rules [10]. The “modified accredited” zone at the time encompassed Alcona, Alpena, Antrim, Charlevoix, Cheboygan, Crawford, Emmet, Montmorency, Oscoda, Otsego, and Presque Isle counties, and those portions of Iosco and Ogemaw counties that are north of the southernmost boundaries of the Huron National Forest and the Au Sable State Forest (Figure 1). 

Between June 2002 and September 2004, 12 cattle farms in Michigan were declared bovine tuberculosis positive by State (Michigan Department of Agriculture) and Federal (USDA/APHIS/Veterinary Services) animal health officials. DNA fingerprinting of the *M. bovis *isolates associated with bovine tuberculosis (TB) on all of these farms was confirmed as the Michigan *M. bovis *strain, first characterized in the state in 1999. Access to collect environmental samples potentially contaminated with *M. bovis *was granted for 11 of the 12 farms. Two additional cattle farms, one identified as bovine TB-positive in 2000 and the other identified in 2001, were also investigated at the request of the farm owners. 

Farm investigations were scheduled within an average of two months of the officially recorded bovine TB-positive date for each of the farms identified between June 2002 and September 2004 (Table 1). One farm was investigated 10 days before the official TB-positive date, and the remaining 10 farms were investigated after their officially recorded TB-positive date. The average time between environmental sampling and the official TB-positive date was 56.18 days (average deviation = 27.47; median = 55; max = 107; min = 10). The two farms identified in 2000 and 2001 were investigated 929 days and 612 days, respectively, after the officially recorded TB-positive date for each farm. The cattle from 9 of the 10 farms identified as TB-positive between 2002 and 2004 were depopulated. The investigation and sampling of the TB-positive farms was accomplished before the cattle were depopulated on five farms and after the cattle were depopulated for the remaining 4 farms (Table 1). The average time between farm sampling and cattle depopulation was 32.67 days (average deviation = 11.56; median = 31; max = 55; min = 7). 

**Figure 2:**
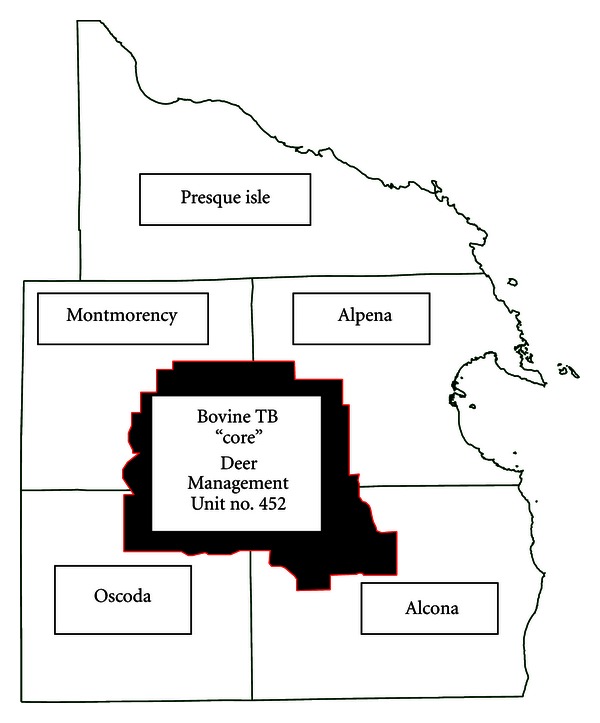
Map of the bovine TB “core” area within the counties of Montmorency, Alpena, Oscoda, and Alcona, Michigan.

Wildlife areas selected for sampling were all within the 5 Michigan townships with the highest apparent prevalence of bovine TB in white-tailed deer. The wildlife sites sampled were either the capture locations of *M. bovis*-infected small mammals, primarily raccoons (*Procyon lotor*) and opossums (*Didelphis virginiana*) or areas of known white-tailed deer congregation. The areas of known white-tailed deer congregation selected were all located in the region designated as the “core” of the endemic area of bovine TB currently affecting white-tailed deer [6]. The “core” is defined by the administrative boundaries of the Michigan Department of Natural Resources Deer Management Unit number 452 (Figure 2).

### 2.2. Sampling Site and Substrate Selection

**Figure 3:**
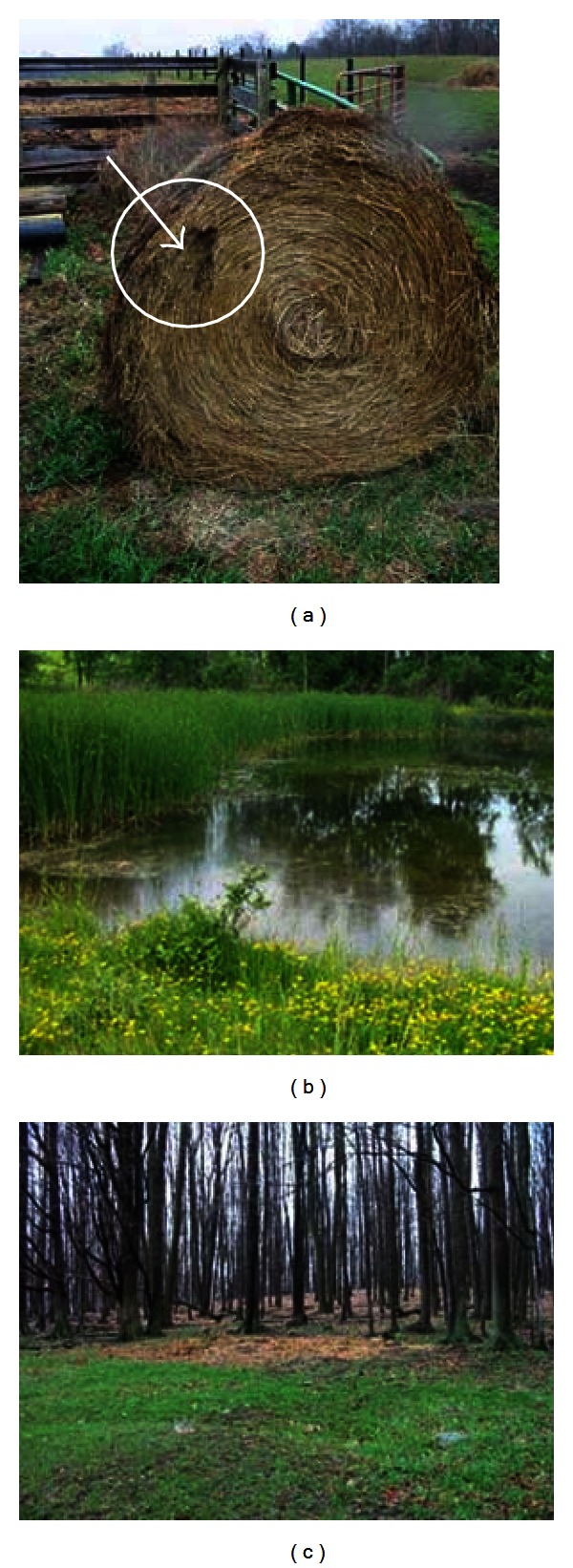
Three photographs of sampling locations selected during investigations of bovine tuberculosis-positive cattle farms: (a) an unprotected hay bale with evidence of deer feeding activity; (b) a pond in a pasture to which both deer and cattle have access; (c) an example of feeding cattle hay on the ground in the woods.

Specific substrates targeted for collection within the sites were selected based on environmental and farm management factors identified as risk factors for bovine TB infection on Michigan cattle farms [12] and previous evaluations of the practice of supplemental feeding of white-tailed deer [1, 8, 13]. On cattle farms, a structured questionnaire with farm managers and a farm walk-through were used to identify approximately 20 sampling locations per farm. Targeted sites included those with evidence of animal concentration (mixed, and single species), feed and water sites with open access to livestock and wildlife, the location within the farm of the infected cattle if known (i.e. pasture, pen or stall), and sites of wildlife observations within the farm borders, including pastures and woodlots. Substrates from the specified locations selected for sampling included feed (hay, grain and silage), pasture grass, soil, fecal material, bedding, and water. Figure 3 depicts three sites selected for sampling on bovine TB-positive farms. 

**Figure 4:**
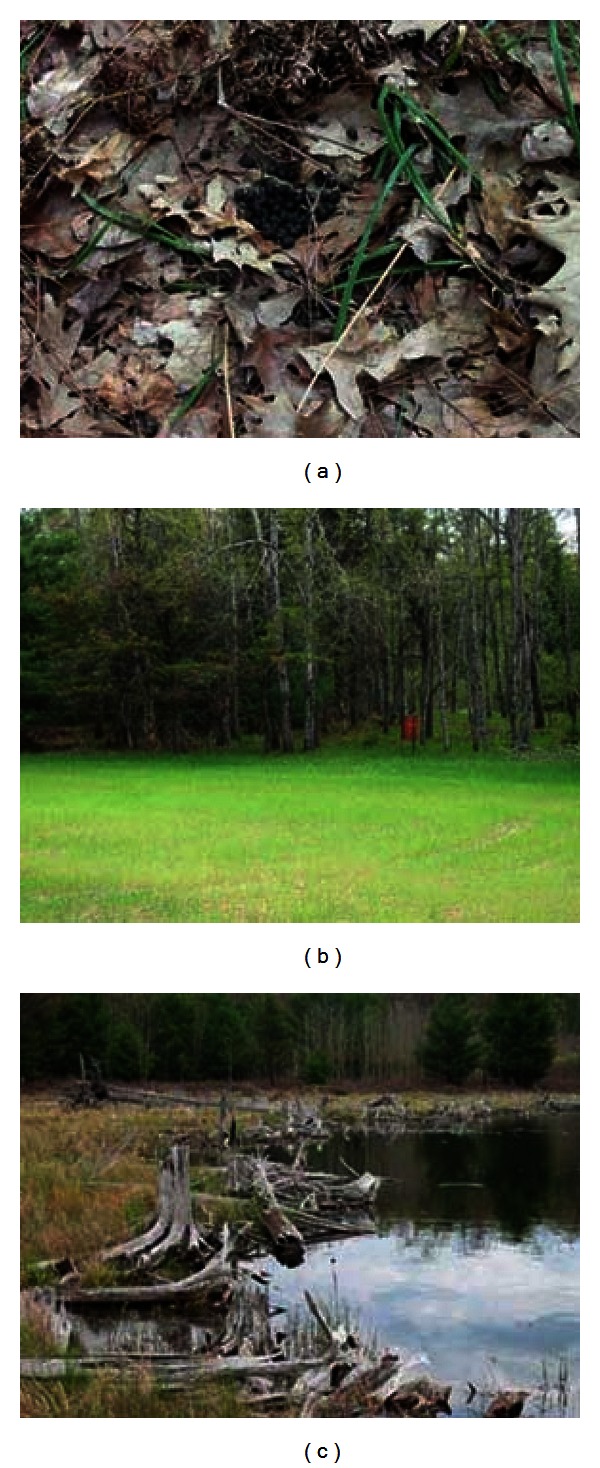
Three photographs of sites selected for sampling in the wildlife bovine TB transmission areas: (a) an oak forest where deer fecal pellets were collected; (b) a deer feeder and a plot of forage planted for deer; (c) a pond near the trap location of a bovine TB-positive (opossum or raccoon).

 The locations for environmental sampling within the wildlife areas selected as potential bovine TB transmission sites were identified with the assistance of wildlife biologists from the Michigan Department of Natural Resources (MDNR) and United States Department of Agriculture (USDA), Animal Plant Health Inspection Service (APHIS), Wildlife Services (WS). Deeryards (naturally sheltered areas used by deer during severe winters with significant snow fall), deer feeding sites, and adjacent areas of open water were selected within the townships identified as having a high prevalence of bovine TB. The area surrounding the trap location of small mammals, primarily raccoons and opossums, identified as TB-positive was surveyed to identify specific substrates for sampling. Substrates selected for sampling from both white-tailed deer and small mammal sites included fecal material, soil, vegetation, and water. Figure 4 depicts three sites selected for sampling in the wildlife bovine TB transmission areas. 

### 2.3. Sample Collection

Approximately 500 grams of substrate was collected from each of the sampling locations identified. Disposable latex gloves or a cleaned and betadine-disinfected shovel was used to collect each sample to prevent cross-contamination. Water samples were collected in 0.5 liter sterile plastic bottles and capped. All other substrates were placed in large capacity Whirl-Pak bags and sealed. The sample containers were labeled with a unique identification number. Additional data corresponding to the sample identification number and a description of the sampling site were collected and recorded on field data sheets. Recorded data included a description of the sample collected, a description and the GPS coordinates of the sample collection location, and a digital photograph of the sampling site. Samples were stored in an insulated cooler surrounded with cold packs. They were transported by vehicle to the Biosafety Level III laboratory at Michigan State University within 8 hours of collection. The samples were stored at 4°C for 12 hours before processing.

### 2.4. Sample Processing

All samples were processed using procedures developed for processing environmental samples for mycobacterial culture that maximized *M. bovis *recovery rates and minimized the level of culture contamination with competing organisms [14]. The procedure developed incorporated the use of CB-18 TB Culture Kit with Lytic Decon II (Integrated Research Technology, LLC, Quest Diagnostics Inc., Baltimore, Md, USA). The CB-18 TB Culture Kit with Lytic Decon II (Integrated Research Technology, LLC) is a commercially available set of reagents and instructions for processing specimens for the detection of mycobacteria by culture. The kit contains C_18_-Carboxypropylbetaine (CB-18), a zwitterionic detergent that replaces NaOH acid wash in the decontamination step and is also thought to decrease surface tension and counteract the natural buoyancy of mycobacteria and facilitate the more efficient collection of the bacilli [15]. The kit also contains the components of a resuspension buffer with lecithin and a mixture of lytic enzymes (lysozyme, zymolyase, *Cytophaga, *and *Trichoderma *extracts). The resuspension buffer is added to the sample sediment before the inoculation of mycobacteria isolation media to reduce contamination with competing organisms [16].

A series of studies using typical environmental substrates (soil, hay, and water) experimentally inoculated, or spiked, with the Michigan strain of *M. bovis *were performed in the mobile Biosafety Level III (BL3) Laboratory on the campus of Michigan State University (MSU) in advance of the investigations of bovine TB transmission sites described in this paper to develop the procedures for processing environmental samples for mycobacterial culture. The minimum detection level for the sample processing procedures developed was determined to be 120 colony forming units (CFUs) of *M. bovis *present in the volume of environmental substrate (5 gm of solid substrates and 7.5 mL of water) processed in the experiments [14]. This volume of substrate was used as the standard volume of substrate processed from environmental samples collected from the field. The experimental studies performed to develop the procedures for processing environmental samples also recorded the rates of mycobacterial culture contamination with competing organisms in *M. bovis *spiked samples processed with conventional NaOH methods as compared to those presented in this paper which incorporate the CB-18 TB Culture Kit with Lytic Decon II. The odds of contamination in NaOH-processed *M. bovis *inoculated environmental substrates that were not pre-sterilized were 11 times that of the same substrates processed with the CB-18 method (OR = 11.0; 95% C.I. (3.2, 37.9)) [14].

In the BL3 Laboratory, environmental samples collected during investigations of bovine TB transmission sites were thoroughly mixed by shaking or swirling the contents within their original sample collection containers and approximately 5 gm of the solid substrates and 7.5 mL of water were transferred for further processing. Soil and fecal samples were placed in individual 7.6 cm × 17.8 cm Whirl-Pak bags. Feed and vegetation samples were chopped with scissors when necessary and placed in individual sterilized Ball pint (473 mL) regular mason jars. Water samples were transferred to individual 50 mL conical centrifuge tubes. 

Sterile water (7.5 mL) and 5 mL of liquefaction solution (trisodium citrate dehydrate and N-acetyl-L-cysteine or NALC) were added to the solid substrates. Samples were then pulverized and homogenized by placing the Whirl-Pak bags in a Stomacher 80 laboratory blender for 30 seconds, and securing a blade unit and gasket on the jars, inverting and blending them for 30 seconds on high with a household blender. Five mL of liquefaction solution were added to the water samples, and they were mixed on high for 30 seconds with a vortex machine. 

The samples were placed upright and allowed to settle for 30 minutes. The top 5 mL of fluid from each sample was removed and transferred to a 50 mL conical tube containing 10 mL of Decontamination Solution (20X Tris-citrate Buffer, CB-18 Stock, or NALC and water). Samples were mixed with a vortex machine and incubated at 37°C for 75 minutes. Sterile water was added to the 50 mL mark on each tube, mixed and centrifuged at 3,000 g for 20 minutes. Pellet-containing tubes were decanted completely. A pipette was used to remove all but 1–3 mL of liquid from samples without a visible pellet. The pellet was resuspended in the supernatant backwash. One mL of sterile water was added and mixed. A 0.5 mL sample was transferred to a 2.0 mL labeled cryogenic vial and frozen at –80°C. One mL of 2X Resuspension Solution (10X-Enzyme Stock-*Trichoderma harzianum* extract, lysozyme and *Lysobacter *extract and NALC) was added to each sample, and they were incubated for 45 minutes at 37°C.

### 2.5. Mycobacterial Culture and Isolation

CB-18-processed samples were inoculated onto solid media slants and plates containing modified Middlebrook 7H11 agar (Becton-Dickinson) with sodium pyruvate (Diagnostic Center for Population and Animal Health, Lansing, Mich, USA) and 7H11 Selective plates (Becton-Dickinson). Solid media slants and plates were incubated at 37°C for 8–12 weeks and examined weekly for colony formation. Positive mycobacterial cultures and colonies on solid media were subjected to an acid-fast smear analysis to confirm the presence of acid-fast bacteria using standard protocols for slide preparation, staining, and examination [17]. Acid-fast-positive isolates were identified to the *Mycobacterium tuberculosis *complex species group using a genetic probe (AccuProbe, Gen-Probe, San Diego, Calif, USA). Biochemical tests and high-performance liquid chromatography were performed by the Michigan Department of Community Health (MDCH) Tuberculosis/Mycology Laboratory to speciate non-*M. tuberculosis* complex mycobacteria or to differentiate between *Mycobacterium bovis *and other members of the *M. tuberculosis *complex.

### 2.6. Statistical Analysis

Excel spreadsheets and statistical functions (AVERAGE, AVEDEV, MEDIAN, MAX, and MIN) were used to generate simple descriptive statistics characterizing the bovine tuberculosis transmission sites and the specific locations identified as sites of potential environmental contamination with *Mycobacterium bovis *(Excel, Microsoft Office XP Professional). Excel spreadsheets were also used to record mycobacterial culture results and summarize the data on the isolation of acid-fast bacteria and the presence or absence of contamination with competing organisms. Comparisons between mycobacterial culture result, substrate type, and presence or absence of contamination with competing organisms were made using the Cochran-Mantel-Haenszel test for *n*-way cross-tabulation tables and the SAS software program (Proc Freq; Tables/CMH, SAS version 9.0, Cary N.C.: SAS Institute, Inc.).

## 3. Results

### 3.1. Cattle Farms: General Characteristics and Farm Management Practices

Of the thirteen bovine tuberculosis cattle farms investigated, four were dairy operations, seven were beef cow/calf operations, one was a small beef feeder operation, and one was a combination cow/calf and feeder operation. The number of adult cattle on the farms ranged from 14 to 239 and averaged 74. The average size of the farm properties was 251 acres. Only 1 farm reported fence-line contact with another cattle farm. Approximately half of farms that were identified as bovine TB-positive between June 2000 and September 2004 purchased 100% of their cattle from outside sources. The majority of the farms did not raise any other kind of livestock, with the exception of chickens, and all but one reported the presence of pet dogs and cats on the farm (Table 2).

Full farm investigations (interviews with farm owners or primarily cattle managers and farm walkthrough) were performed on the first 12 farms sampled. USDA/APHIS/WS personnel collected environmental samples from the 13th farm, but only very general farm characteristics were recorded. Although 10 of the 12 farms had cattle housing (barn, feedlot, or barn yard) facilities, cattle spent more than 50% of their time outside on 9 of the 12 farms. The three farms on which cattle spent more than 50% of their time inside were dairy herds. Only 1 farm, a dairy, never fed cows outside. Cattle on all of the farms had access to water sources outside, and on 10 of the farms cattle had access to surface water (ponds, streams, or water ways). Hay was provided to cattle in a feeder on 4 of the farms, but it was also provided on the ground on 2 of these. Ten of the 12 farms provided hay to their cattle outside on the ground. A summary of the cattle management practices on each of the farms is summarized in Table 3(a). 

An examination of feed storage and general fencing practices on the farms revealed that hay (round bales) were stored outside and unprotected on 8 of the 12 farms. Round bales of hay were stored in the fields, along fencerows or in the woods on 4 of the farms. Five of the farms used some barbed wire, and 9 of the farms used electric fencing. Two of the farms had “deer proof” fencing around their hay that had been installed through a USAID/APHIS/WS program after their cattle were identified as bovine TB-positive. A summary of the fencing and feed storage practices is provided in Table 3(b).

All of the farmers interviewed reported observing deer on their property. All but one of the 12 farms reported observing deer in their cattle pastures, and all 12 farms reported observing deer feeding on either their pastures or crop fields. Deer were not observed drinking water from outdoor tanks but 5 of the farms reported observing evidence of deer using the same open water sources accessible to cattle on the farm. Six of the 12 farms reported observing deer feeding on harvested hay intended for cattle, and all but 1 farm reported the presence of land features preferred by deer (orchards or cedar swamps) on or adjacent to their property (Table 3(c)). Producers on all farms surveyed reported observations of wildlife other than deer. The species observed included raccoons (*Procyon lotor*), opossum (*Didelphis virginiana*), red fox (*Vulpes vulpes*), coyote (*Canis latrans*), turkeys (*Meleagris gallopavo*), striped skunks (*Mephitis mephitis*), bobcats (*Felis rufus*), black bears (*Ursus americanus*), and porcupine (*Erethizon dorsatum*). 

### 3.2. Cattle Farms (Samples Collected)

A total of 409 samples were collected from 13 farms. Approximately 20 sites were selected for sampling on each farm. One sample was collected per site for the first 10 farms. An effort was made to intensify sampling on the final two farms and more samples were collected per site. A total of 140 samples were collected from farm 112, and a total of 52 samples were collected from farm 113 (Table 4).

### 3.3. Wildlife Areas (Samples Collected)

A total of 97 samples were collected from 5 wildlife areas. Approximately 20 locations were selected for sampling in each wildlife area, and approximately 1 sample was collected per site. An attempt was made to distribute the sampling across substrate type (Table 5).

#### 3.3.1. *Mycobacterium bovis* Culture Results

None of the samples collected from the bovine TB-positive farms were positive for *Mycobacterium bovis* based on mycobacterial culture. A number of acid-fast organisms were isolated but further testing revealed that none of these were members of the *M. tuberculosis* complex (Table 4). Acid-fast organisms were most commonly isolated from soil, vegetation, manure, and cattle feed samples, but an association between the isolation of acid-fast bacteria and sample type was not found (Mantel-Haenszel *X^2^* = 0.04; degree freedom = 1; *P* value = 0.84). Samples processed from wildlife areas produced similar results. Acid-fast bacteria were isolated from samples of soil and vegetation, but no significant associations between substrate type and acid-fast bacterial isolation were found. The isolates of the non-*M. tuberculosis *complex acid-fast bacteria that could be identified to species included *M. fortuitum*,* M. avium, M. fortuitum-chelonae*, and *Mycobacterium sp.* Group IV*.*

The prevalence of contamination (overgrowth of the cultures with mold and nonmycobacteria) was high. Twenty-seven percent of the samples collected from bovine TB-positive farms (Table 4) and 11% of the samples collected from wildlife areas (Table 5) were contaminated. Soil samples and substrates mixed with manure were most likely to be contaminated, but no significant association between sample substrate and the presence of contamination was found (Mantel-Haenszel *X* ^2^ = 0.01; degree freedom = 1; *P* value = 0.92).

## 4. Discussion

*Mycobacterium bovis*, the causative agent of bovine TB, continues to circulate among cattle and white-tailed deer in Michigan. Over the two-year span of this study, from June 2002 until September 2004, 12 cattle farms in northern Lower Michigan were identified as bovine TB-positive (Michigan Department of Agriculture). The cattle herds identified as bovine TB-positive during this period were all in the USDA designated “Modified Accredited Zone” (Figure 1) where annual whole-herd bovine TB testing is required [10]. The farms identified, therefore, likely represent new bovine TB infection and relatively recent transmission events. The estimated prevalence of *M. bovis *within these herds was low and no evidence of disseminated disease in individual cattle was found, further supporting a relatively recent exposure to *M. bovis*. Similarly, on-going surveillance for bovine TB in the white-tailed deer population during the same period revealed an apparent prevalence fluctuating around 2.0% in animals originating in MDNR, Deer Management Unit no. 452, the endemic focus of bovine TB in white-tailed deer in the State (Figure 2) [9]. A number of the bovine TB-positive white-tailed deer identified during this routine surveillance were yearlings, indicating new infection and recent bovine TB transmission events. 

It is generally accepted that *M. bovis *is transmitted within deer populations through a combination of direct and indirect means and within cattle herds, primarily through close contact and direct routes of disease transmission [18, 19]. Interspecies transmission of bovine TB between white-tailed deer and cattle, however, likely occurs primarily through indirect routes of transmission since little evidence of direct contact between the species exists [7]. Despite evidence of on-going bovine TB transmission in northeast Michigan, this study failed to isolate *Mycobacterium bovis *from environmental substrates collected from bovine TB-positive farms and wildlife areas.

### 4.1. Potential Sites of *M. bovis* Contamination of the Environment

#### 4.1.1. Bovine TB-Positive Farms

The general characteristics of the 13 bovine TB-positive farms selected for investigation and environmental sampling in this study were similar to those of the initial group of bovine TB-positive farms identified between 1998 and 2002 [3]. The majority of the farms were small beef cattle operations, and they were all located in northeast lower Michigan. Particular cattle management practices that have been identified as risk factors associated with tuberculosis on cattle farms in northeast Michigan in the past [12] and those that would facilitate the indirect transmission of bovine TB from deer to cattle via *M. bovis *contaminated substrates included (1) maintenance of cattle outside for more than 50% of the time outside (75% of farms); (2) feeding cattle outside (92%) and feeding cattle outside exclusively (58%); (3) watering cattle outside with access to open water (streams, ponds, etc.) (83%); (4) feeding cattle hay on the ground (83%). 

The practices outlined above are only a bovine TB risk to cattle if infected white-tailed deer in the area also have access to the hay, pasture, and water sources identified as cattle feeding and watering sites. Answers to “deer incursion” survey questions indicated that deer were seen on the premises of 100% of the farms identified. Ninety-two percent of respondents observed deer in pastures, 50% observed evidence of deer feeding on hay intended for cattle, and 42% observed evidence of deer drinking from open water sources on the farms. Electric (75%) and barbed wire (42%) fencing was used on these cattle farms, but feed was only stored in “deer proof” facilities on 25% of the farms surveyed. 

Dairy cattle operations generally maintained their cattle inside housing more than 50% of the time, fed their cattle inside, and used hay feeders; however, cattle had access to open water and hay was stored outside and unprotected on many of the premises investigated.

#### 4.1.2. Wildlife Bovine TB Transmission Sites

The investigations of wildlife areas were performed in the summer and spring. In the spring and summer months, particular wildlife management practices that would facilitate the indirect transmission of bovine TB were not observed with the exception of the identification of small fields in wooded areas planted with grass forages to attract deer and a limited number of empty deer feeding stations which would likely have been active in winter.

### 4.2. Detection of *M. bovis* in the Environment: Agent-Related Factors

Properties of *M. bovis *contribute to difficulties associated with isolating the organism from environmental substrates. *M. bovis *is particularly difficult to culture. The necessity of a bactericidal decontamination step, coupled with the cording behavior and natural buoyancy of *M. bovis*, reduces the success of mycobacterial culture methods [17, 20]. The additional presence of large numbers of saprophytic bacteria, molds, and other infectious organisms in environmental samples further interferes with the sensitivity of detection of *M. bovis* by bacterial culture. Attempts were made to improve the success of isolating *M. bovis *with bacterial culture methods by processing specimens with CB-18 TB Culture Kit with Lytic Decon II (Integrated Research Technology, LLC, Quest Diagnostics Inc., Baltimore, Md, USA). Although *M. bovis *was not isolated, other mycobacterial species were successfully identified from environmental substrates collected suggesting that the techniques used were capable of detecting mycobacterial species from environmental samples in the presence of other competing microbes. 

Culture contamination, an overgrowth of mold and nonmycobacterial species, affected our attempts to isolate *M. bovis *from approximately 25% of the samples collected. Although no significant associations were found between contamination rates and sample substrate type, the distribution of contamination in samples suggested that soil, vegetation samples, and those mixed with cattle manure were more likely to produce contaminated mycobacterial culture results. These data indicate that the sensitivity of isolation of *M. bovis *from these particular substrates types may be further reduced. 

The challenges associated with detection of *M. bovis *in environmental substrates has been cited as a potential cause of failure to isolate *M. bovis *from environmental substrates from other areas identified as sites of natural transmission of bovine TB in Michigan. These include a captive white-tailed deer facility [21] and one of the first cattle farms identified as TB positive in the current outbreak of *M. bovis *in Michigan (Kaneene, personal communication). *M. bovis* has been isolated from environmental substrates contaminated in the course of experimental transmission studies among deer [22, 23] and between deer and cattle [24]; however, even under these “ideal” circumstances, success has been intermittent. 

### 4.3. Detection of *M. bovis* in the Environment: Host Related Factors

One of the limitations of opportunistic environmental sampling of bovine TB-positive cattle herds and identified wildlife bovine TB transmission areas is that a time lag likely exists between the time at which the *M. bovis *shedding animal (cattle or deer) was present on the premises and the time of sample collection. This time lag is likely exacerbated by intermittent shedding of *M. bovis *from both infected deer [23] and infected cattle [25, 26]. The probability of collecting an environmental sample from an identified bovine TB transmission site in the time period the infected animal is present and at a time when it is shedding *M. bovis *may be very low. 

The denning behavior and patterns of movement of other wildlife reservoirs of bovine TB, primarily brushtail possums (*Trichosurus vulpecula*) in New Zealand [27] and European badgers (*Meles meles*) in Ireland and Great Britain [28, 29], allow for a closer approximation of the opportunities for their potential direct and indirect contact with cattle on bovine TB-affected farms. Although somewhat predictable, free-ranging white-tailed deer have much larger home ranges and their presence on cattle farms is more transient [30]. The behavior of potentially bovine TB-infected white-tailed deer does not allow for a fine level of targeted sampling of environmental substrates for the detection of *M. bovis*. 

### 4.4. Detection of *M. bovis* in the Environment: Landscape-Related Factors

Efforts to isolate *M. bovis* in other regions have yielded similar results when samples were collected under natural disease transmission conditions [31]. Researchers interested in the persistence of *M. bovis *in the environment have turned to experimental inoculation studies and an assessment of the conditions that support or inhibit *M. bovis *survival [27, 32–39]. This is due primarily to the difficulty of identifying the exact location of *M. bovis *contamination over what is often a very large potentially contaminated site. This study faced the same challenge. Financial and time constraints limited the number and volume of environmental substrates that could be collected and processed from sites potentially contaminated with *M. bovis*. These constraints limited the total surface area of both bovine TB-positive farms and wildlife areas that could be sampled effectively. 

This field investigation of bovine TB transmission sites confirmed the findings of earlier studies that have identified environmental and cattle farm management practices [12] in northeast Michigan that may facilitate indirect interspecies transmission of bovine TB between deer and cattle. Investigations of wildlife TB transmission areas also produced evidence of deer feeding and baiting practices that have been identified by other authors as likely contributing to the indirect transmission of bovine TB among white-tailed deer [1, 8, 9, 13]. The failure to isolate *M. bovis *from environmental substrates collected from bovine TB-positive cattle farms and wildlife areas was likely due to agent, host, and landscape factors that contribute to the difficulty of identifying specific sites of *M. bovis *contamination and recovering *M. bovis *from environmental substrates.

## Figures and Tables

**Table 1:** Time lag between farm investigation (environmental sampling), official TB-positive date, and date of cattle depopulation. Farms identified as TB+ in 2000 and 2001 (nos. 105 and 106) were not included in this analysis.

Farm number	TB+ date	Depopulation date	Sampling date	TB+ to sampling days	Depopulation to sampling days
101	09/20/02	10/02/01	09/10/02	−10	−22
102	07/17/02	09/17/02	09/10/02	55	−7
103	07/16/02	08/21/02	09/13/02	59	23
104	06/12/02	10/29/02	09/27/02	107	−32
107	01/09/03	03/31/03	02/28/03	50	−31
108	11/27/02		03/04/03	97	Not depopulated
109	01/27/03	03/11/03	05/05/03	98	55
110	05/27/03	06/03/03	07/02/03	36	29
111	11/10/03	01/21/04	12/02/03	22	−50
112	12/23/03		03/03/04	71	Not depopulated
113	08/20/04	07/19/04	09/02/04	13	45

			Average:	56.18 days	32.67 days

**Table 2:** General characteristics of bovine TB-positive farms selected for environmental sampling.

Farm number	Farm type	Herd size	Farm size	% Purchased	Fence line contact	Other livestock	Pets
		No. Adults	Acres				Dogs/Cats
101	Beef feeder	31	110	100	No	No	Yes
102	Dairy	79	428	0	No	No	Yes
103	Cow/calf	37	102	100	No	No	Yes
104	Cow/calf	40	128	100	No	No	No
105	Cow/calf	108	330	- - - - -*	Yes	No	Yes
106	Cow/calf	103	540	- - - - -*	No	Horses	Yes
107	Cow/calf	23	188	5	No	Chickens	Yes
108	Dairy	239	500	100	No	No	Yes
109	Cow/calf	19	80	>50	No	Chickens	Yes
110	Cow/calf	48	300	25	No	Horses/sheep	Yes
111	Cow/calf-feeder	14	244	<5	No	Chickens	Yes
112	Dairy	68	65	<5	No	No	Yes
113	Dairy	148					

	Average:	73.62	251.25				

*Farm 105 and 106 were TB positive before the study period.

**Table:** (a)

A	Housing barn/lot	>50% outside	Feed outdoors	Feed outdoors only	Hay feeder	Hay ground	Water tank outdoors	Water open outdoors
% yes:	83%	75%	92%	58%	33%	83%	50%	83%

**Table:** (b)

B	Hay unprotected outdoors	Round bale field/fence/woods	Feed fenced “deer proof”	Cattle fencing barbed	Cattle fencing electric
% yes:	67%	33%	25%	42%	75%

**Table:** (c)

C	In pasture	Near cattle housing	Near home	Drinking tank water	Drinking open water	Feeding on pasture/crop	Feeding on hay	Deer preferred habitat
% yes:	92%	25%	42%	0%	42%	100%	50%	83%

**Table 4:** Mycobacterial culture results of environmental substrates collected from bovine tuberculosis-positive farms.

	Total	AFB + (%)	Contamination + (%)
Pasture and vegetation	79	13 (16%)	15 (19%)
Soil	75	13 (17%)	32 (43%)
Open water	78	2 (3%)	12 (30%)
Hay	50	5 (10%)	10 (20%)
Cattle feed	10	3 (30%)	2 (20%)
Barn water	4	0 (0%)	0 (0%)
Bedding	22	0 (0%)	9 (41%)
Manure	40	1 (3%)	12 (30%)
Manure mix	23	6 (26%)	12 (52%)
Deer feces	10	0 (0%)	4 (40%)
Wildlife feces	17	1 (6%)	3 (18%)
Bear hair	1	0 (0%)	0 (0%)

	409	44 (11%)	111 (27%)

**Table 5:** Mycobacterial culture results of environmental substrates collected from potential wildlife bovine tuberculosis transmission sites.

	Total	AFB + (%)	Contamination + (%)
Pasture and vegetation	17	1 (6%)	4 (24%)
Soil	24	2 (8%)	5 (21%)
Open water	22	0 (0%)	0 (0%)
Wildlife feces	5	0 (0%)	1 (20%)
Deer feces	28	0 (0%)	0 (0%)
Grain	1	0 (0%)	1 (100%)

	97	3 (3%)	11 (11%)
